# Preoperative stents for the treatment of obstructing left-sided colon cancer: a national analysis

**DOI:** 10.1007/s00464-022-09650-8

**Published:** 2022-10-11

**Authors:** Joseph Hadaya, Arjun Verma, Yas Sanaiha, Russyan Mark Mabeza, Formosa Chen, Peyman Benharash

**Affiliations:** 1 Department of Surgery, David Geffen School of Medicine at UCLA, Los Angeles, CA USA; 2 Department of Surgery, Olive View-UCLA Medical Center, Sylmar, CA USA

**Keywords:** Large bowel obstruction, Colonic stenting, Colonic obstruction, Colonic decompression, Colectomy

## Abstract

**Background:**

Given the risks associated with urgent colectomy for large bowel obstruction, preoperative colonic stenting has been utilized for decompression and optimization prior to surgery. This study examined national trends in the use of colonic stenting as a bridge to resection for malignant large bowel obstruction and evaluated outcomes relative to immediate colectomy.

**Methods:**

Adults undergoing colonic stenting or colectomy for malignant, left/sigmoid large bowel obstruction were identified in the 2010–2016 Nationwide Readmissions Database. Patients were classified as immediate resection (IR) or delayed resection (DR) if undergoing colonic stenting prior to colectomy. Generalized linear models were used to evaluate the impact of resection strategy on ostomy creation, in-hospital mortality, and complications.

**Results:**

Among 9,706 patients, 9.7% underwent colonic stenting, which increased from 7.7 to 16.4% from 2010 to 2016 (*p* < 0.001). Compared to IR, the DR group was younger (63.9 vs 65.9 years, *p* = 0.04), had fewer comorbidities (Elixhauser Index 3.5 vs 3.9, *p* = 0.001), and was more commonly managed at high-volume centers (89.4% vs 68.1%, *p* < 0.001). Laparoscopic resections were more frequent among the DR group (33.1% vs 13.0%, *p* < 0.001), while ostomy rates were significantly lower (21.5% vs 53.0%, *p* < 0.001). After risk adjustment, colonic stenting was associated with reduced odds of ostomy creation (0.34, 95% confidence interval 0.24–0.46), but similar odds of mortality and complications.

**Conclusion:**

Colonic stenting is increasingly utilized for malignant, left-sided bowel obstructions, and associated with lower ostomy rates but comparable clinical outcomes. These findings suggest the relative safety of colonic stenting for malignant large bowel obstruction when clinically appropriate.

**Graphical abstract:**

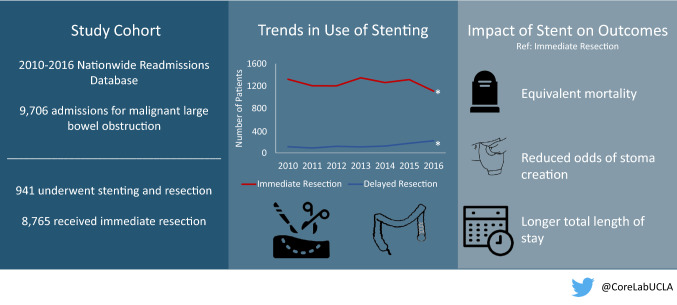

**Supplementary Information:**

The online version contains supplementary material available at 10.1007/s00464-022-09650-8.

Colonic malignancies are the second leading cause of cancer-related mortality, with over 50,000 attributable deaths annually in the USA alone [1]. Despite advances in diagnostic modalities and improved screening for colon cancer, malignant large bowel obstruction occurs in roughly 15% of patients with colon cancer in the USA [2, 3]. The majority of malignant large bowel obstructions are secondary to large left-sided tumors and require urgent management [4, 5].

Emergent colectomy for acute obstruction is associated with significant risk of mortality, while complications are reported in 37–45% of patients [6, 7]. Ostomy rates as high as 65%, with 26–53% reported to be permanent, negatively impact quality of life and contribute to increased readmissions and resource utilization in this population [8, 9]. Several small randomized trials and retrospective series have reported on the utility of colonic stenting as part of the treatment algorithm for malignant left-sided colonic obstructions. A meta-analysis by De Ceglie et al. evaluated 822 patients with left-sided malignant large bowel obstruction (LBO) in 14 studies and found an overall reduction in ostomy use despite significant heterogeneity among the studies [10]. Despite conflicting results from several initial randomized trials, two of which required early termination for safety concerns, contemporary studies have demonstrated the efficacy of colonic stenting, with low rates of rates of iatrogenic complications [11, 12]. Similarly, a recent meta-analysis demonstrated similar long-term oncologic outcomes for patients undergoing emergency surgery or colonic stenting as a bridge to surgery, when performed with curative intent [13]. Therefore, the European Society of Gastrointestinal Endoscopy and the American Society of Colon and Rectal Surgeons currently recommend discussion and shared decision-making regarding the use of colonic stents in patients with obstructing left-sided colon cancer. [14]

Given the limited sample size of existing trials, the present work examined a nationally representative cohort of patients with malignant bowel obstruction to evaluate outcomes associated with colonic stenting as a bridge to resection. We compared clinical outcomes and ostomy rates for patients undergoing immediate colonic resection with those undergoing delayed colectomy after bridging. We hypothesized lower ostomy rates, but otherwise similar clinical outcomes with the use of preoperative stenting.

## Materials and methods

### Data source and study groups

The present study was a retrospective cohort study using the 2010–2016 Nationwide Readmissions Database (NRD). Maintained by the Agency for Healthcare Quality and Research as part of the Healthcare Cost and Utilization Project (HCUP), the NRD is the largest, all-payer, readmissions database and accrues data from 28 individual state inpatient databases [15]. Using robust survey weighting algorithms, the NRD provides estimates for approximately 60% of hospitalizations in the USA. Through patient-specific linkage numbers, patients are tracked across inpatient hospitalizations within each calendar year, thus limiting follow-up to a maximum of 12 months.

**Fig. 1:**
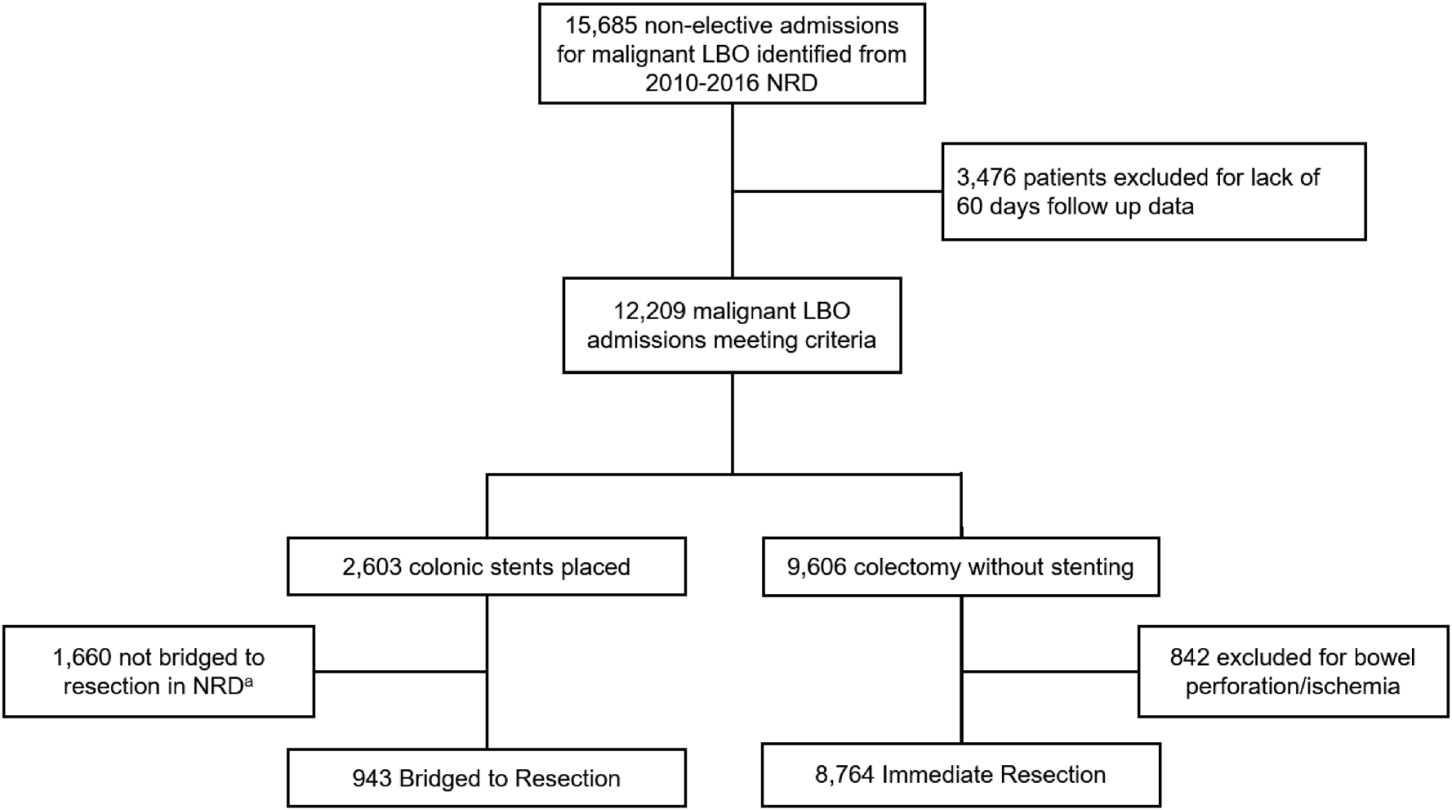
Study flowchart depicting study criteria and analytic groups. ^a^Patients not bridged to resection after colonic stenting may have undergone palliative stenting, undergone surgery at a non-NRD participating hospital, died as an outpatient, or lost to follow-up. DR, delayed resection; IR, immediate resection; LBO, large bowel obstruction; NRD, Nationwide Readmissions Database

All adult (≥ 18 years) patients admitted with a diagnosis of large bowel obstruction and left/sigmoid colon cancer who underwent colectomy (left, extended left, sigmoid, subtotal), diversion, or colonic stenting were identified in the NRD using *International Classification of Disease, Ninth and Tenth Edition* (ICD-9 and ICD-10) codes (Supplemental Table 1). Hospitalizations lacking at least 60 days of follow-up time (90th percentile of time interval between stenting and colectomy on exploratory analysis) were excluded from the study. Additionally, those with missing data for key variables including age, sex, and in-hospital mortality were excluded (*n* = 66, < 1%). Study population and exclusion criteria are presented in Fig. 1. Patients not receiving colonic resection following stenting were not included in analysis as these patients may have either undergone palliative stenting, died in the outpatient setting, underwent surgery in a hospital not captured in the NRD, or did not have sufficient follow-up.

**Table 1:** Baseline characteristics of patients undergoing IR or DR (bridged with colonic stent)

	IR (*n* = 8,764)	DR (*n* = 943)	*P*-value
Age	65.9 ± 14.5	63.9 ± 14.7	0.04
Female	3903 (45.8)	419 (44.4)	0.62
Elixhauser Comorbidity Index	3.89 ± 1.59	3.54 ± 1.57	0.001
*Payer Status*			0.03
Private	2393 (27.3)	330 (35.1)	
Medicare	4560 (52.1)	424 (45.1)	
Medicaid	972 (11.1)	122 (13.0)	
Other Payer^a^	828 (9.5)	65 (6.8)	
*Income Quartile*			0.007
First (Lowest)	2565 (29.8)	223 (24.2)	
Second	2127 (24.7)	196 (21.3)	
Third	2081 (24.2)	234 (25.4)	
Fourth (Highest)	1826 (21.1)	268 (29.1)	
Laparoscopic Approach	1137 (13.0)	312 (33.1)	< 0.001
*Comorbidities*			
Anemia	1235 (14.1)	142 (15.0)	0.69
Coagulopathy	227 (2.6)	27 (2.9)	0.74
Chronic Liver Disease	370 (4.2)	56 (6.0)	0.15
Chronic Lung Disease	1034 (11.8)	81 (8.6)	0.16
Congestive Heart Failure	607 (6.9)	55 (5.9)	0.53
Coronary Artery Disease	931 (10.6)	101 (10.7)	0.95
Electrolyte Disorder	4231 (48.3)	423 (44.8)	0.25
Hypertension	4150 (47.4)	421 (44.6)	0.40
Hypothyroidism	630 (7.2)	67 (6.0)	0.36
Metastatic Disease	1474 (16.8)	171 (18.1)	0.59
Neurologic Disorder	305 (3.5)	15 (1.6)	0.06
Obesity	754 (8.6)	85 (9.0)	0.80
Peripheral Vascular Disorder	467 (5.3)	34 (3.6)	0.19
Pulmonary Circulatory Disorder	257 (2.9)	18 (2.0)	0.21
Renal Failure	540 (6.2)	62 (6.5)	0.82
Weight Loss	1865 (21.3)	162 (17.2)	0.09
*Hospital Bed Size*			0.001
Small	1230 (14.0)	75 (7.9)	
Medium	2183 (24.9)	157 (16.7)	
Large	5351 (61.1)	711 (75.4)	
*Colectomy Volume*^*b*^			< 0.001
First Tertile (Low)	470 (5.4)	< 10	
Second Tertile	2329 (26.6)	94 (9.9)	
Third Tertile (High)	5965 (68.1)	843 (89.4)	
Teaching Hospital	4474 (51.1)	738 (78.3)	< 0.001

Categorical variables reported as count and percentage. Continuous variables reported as mean and standard deviation.

^a^Other payer includes uninsured and self-pay.

^b^Colectomy volume defined into three tertiles based on annual, institutional volume of colon resections

Patients who underwent colonic resection at the index hospitalization without preoperative stenting were considered the *Immediate Resection (IR)* group. The *Delayed Resection (DR)* group encompassed those who underwent colonic stent placement with resection at the index or subsequent hospitalization. Patients with a diagnosis of bowel ischemia or perforation were excluded if they underwent immediate resection, as these patients would not have been considered candidates for colonic stenting.

### Variable definitions and outcomes

Patient and hospital characteristics were defined according to the NRD Data Dictionary and included age, sex, primary insurer (Medicare, Medicaid, private insurance or other), income quartile, hospital bed size and teaching status [15]. The Elixhauser Comorbidity Index, a previously validated composite score encompassing 30 chronic conditions, was used to quantify the burden of chronic disease [16]. Hospitals were divided into low-, medium-, and high-volume tertiles based on the annual institutional volume of colon resections. The development of infectious complications was not able to be ascertained, given significant undercoding of events, such as sepsis [17].

Mortality was defined as death during the hospitalization for stenting or colectomy. Complications were defined using ICD-9 or ICD-10 diagnosis codes (Supplementary Table 1) and grouped into cardiac (ventricular tachycardia, ventricular fibrillation, cardiac arrest), thromboembolic (deep vein thrombosis, pulmonary embolism), and pulmonary (pneumonia, mechanical ventilation > 96 h). Length of stay (LOS) was assessed at the colectomy admission, and cumulatively when colonic stenting and colectomy were performed at separate admissions.

The primary outcome of the study was ostomy creation, while mortality and complications were secondarily considered.

### Statistical analysis

Continuous variables are reported as mean with standard deviation or median with interquartile range for non-normally distributed variables (LOS). Categorical variables are reported as count (*n*) and percentage (%). Chi-squared and adjusted Wald or Wilcoxon rank-sum tests were used for comparisons of categorical and continuous variables, respectively. Temporal trends were assessed using Cuzick’s rank-based non-parametric test [18]. Entropy balancing was used to adjust for differences in baseline characteristics between the IR and DR groups. This approach allows for retention of all observations and obviates the need for specific propensity score models. Entropy balancing has previously been shown to be a robust analytic method in observational and retrospective cohort studies [19, 20]. Following application of entropy balancing weights, generalized linear models were fit to evaluate the independent association between use of colonic stent as a bridge to resection and outcomes of interest. Additional adjustment for covariates was not necessary as this reweighting scheme produces balanced populations. A Gaussian distribution with square root link was used for length of stay. Model performance was assessed using Akaike and Bayesian information criteria. Regression results are reported as estimate with 95% confidence interval (CI).

Power analysis was performed a priori based on the primary outcome to identify the sample size needed to detect a difference between the immediate and delayed resection. An effect size of 20% relative reduction in ostomy rates for DR relative to IR was considered clinically significant, with an *α* = 0.05 and *β* = 0.20.

The Institutional Review Board at the University of California, Los Angeles deemed this study exempt from full review. Statistical analysis was performed using Stata 16.0 (StataCorp, College Station, Texas). Statistical significance was set at an *α* < 0.05.

## Results

### Trends in the use of colonic stents for left and sigmoid malignant large bowel obstruction for patients receiving surgical management

**Fig. 2:**
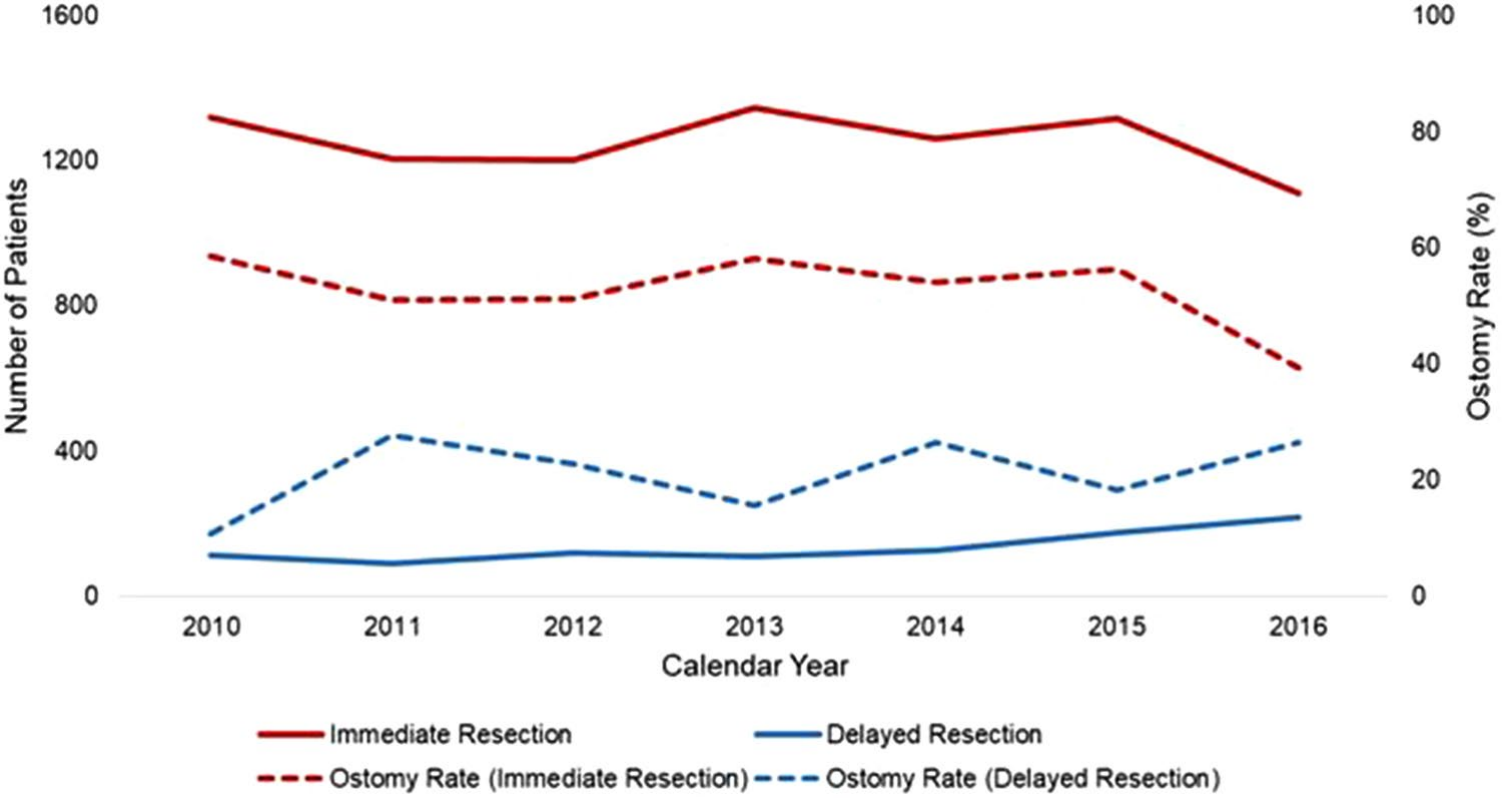
Trends in use of colonic stenting for malignant large bowel obstruction and associated ostomy rates from 2010 to 2016. Colonic stenting as a bridge to resection significantly increased (*p* < 0.001). Ostomy rates for IR significantly decreased (*p* < 0.001), while those for DR remained stable. DR, delayed resection; IR, immediate resection

Of an estimated 9,706 patients meeting study criteria, 9.7% comprised the DR group and underwent colonic stenting prior to resection (Fig. 1). The use of colonic stenting as a bridge to resection increased from 7.7% of cases in 2010 to 16.4% in 2016 (*p* < 0.001, Fig. 2). During the study period, ostomy rates decreased from 58.5 to 39.2% in the IR group (*p* < 0.001), but remained stable for DR (*p* = 0.61, Fig. 2).

### Characteristics of patients undergoing colectomy with or without prior colonic stenting

Baseline characteristics of the immediate and delayed resection groups are reported in Table 1. There were no significant differences in age or sex between the two groups. Patients who underwent delayed resection were more commonly privately insured (35.1 vs 27.3%, *p* < 0.001) and in the highest income quartile (29.1 vs 21.1%, *p* < 0.001). Compared to IR, DR patients had a lower cumulative burden of comorbidities (Elixhauser Comorbidity Index 3.5 ± 1.6 vs 3.9 ± 1.6, *p* < 0.001), although the proportion of most specific comorbidities were similar between the two groups (Table 1). Patients in the DR group were more commonly treated at large, teaching hospitals that were in the highest tertile of colonic resection volume (Table 1).

**Fig. 3:**
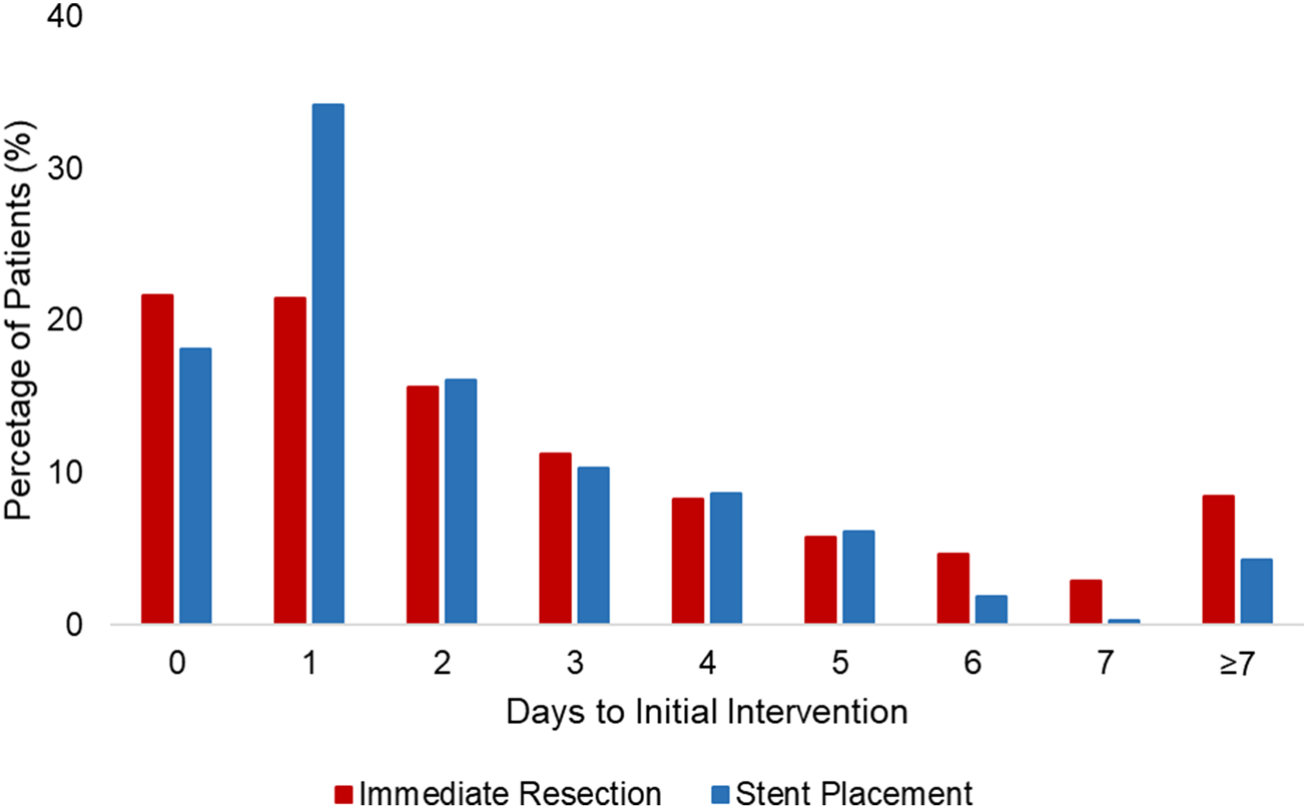
Timing of initial intervention for IR and DR groups. For the IR group, initial intervention was colectomy, while colonic stenting was the initial intervention for the DR group. Time reported as days from admission, with 0 defined as the day of admission. IR, immediate resection; DR, delayed resection

The median time from admission to initial intervention was evaluated for both groups (Fig. 3). The median time from admission to colectomy was 2 days for the immediate resection group, while the median time to colonic stenting was 1 day for those who underwent delayed resection. Of those undergoing delayed resection, 55.1% underwent colectomy at the same admission and 44.9% at a subsequent hospitalization. Specifically, 52.8% underwent colectomy within 1 week, 66.4% within 2 weeks, and 81.4% within 4 weeks of discharge (Fig. 3).

### Unadjusted and adjusted outcomes for delayed resection versus immediate resection

**Fig. 4:**
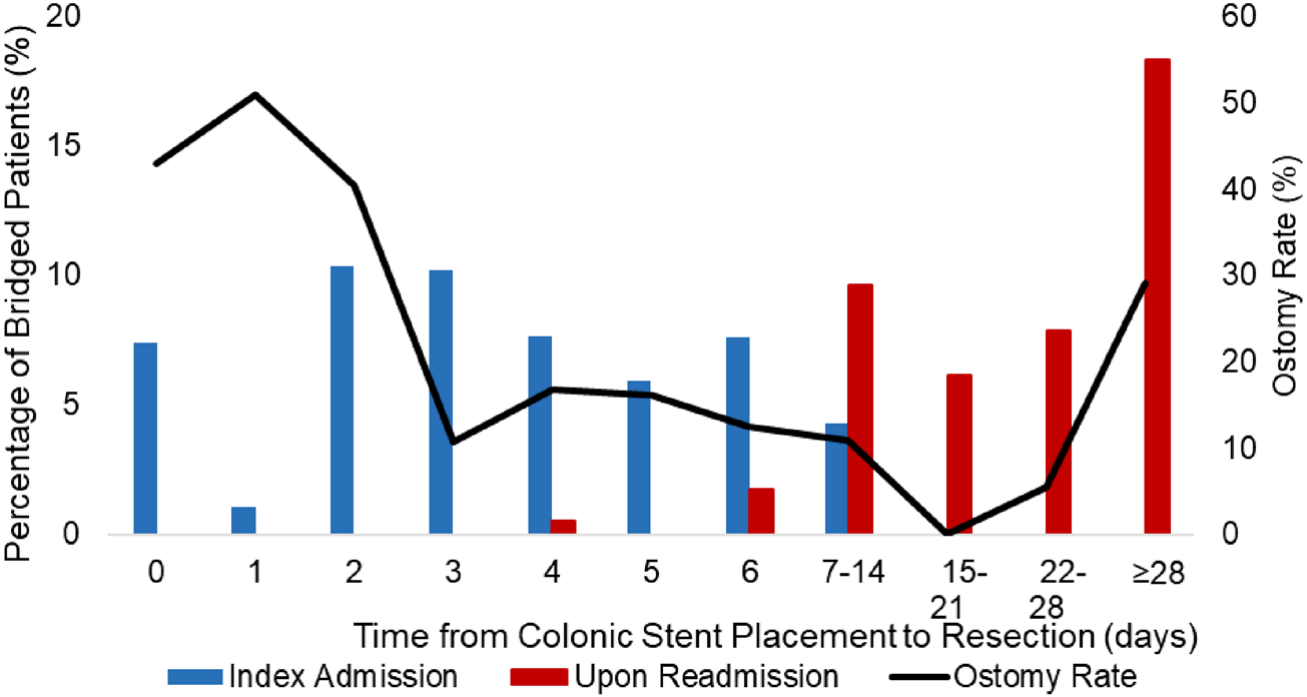
Time from colonic stent placement to resection for the DR group and associated ostomy rates. Colectomy occurred at either the index admission (blue) or subsequent admission (red). A higher rate of ostomy creation was evident for those undergoing resection within 2 days of stent placement (Color figure online)

Unadjusted outcomes for the two groups are reported in Table 2. Compared to 53.0% of the IR group, 21.5% of DR patients received an ostomy (*p* < 0.001). Rates of all studied complications, including pneumonia, mechanical ventilation, aggregate cardiac or thromboembolic events, were similar between the two groups. Unadjusted in-hospital mortality was higher for IR (3.4%) compared to DR (1.2%, *p* = 0.006). Among patients undergoing DR, ostomy rates decreased as time from colonic stent placement to resection increased. Those who underwent resections on day 0, 1, or 2 following stent placement experienced ostomy rates of 40–50%, while patients undergoing colectomy on day 3 experienced rates of 11% (Fig. 4).

**Table 2:** Unadjusted outcomes and for immediate resection vs delayed resection groups

	IR (*n* = 8764)	DR (*n* = 943)	*P*-value
In-hospital mortality	298 (3.4)	12 (1.2)	0.006
Ostomy creation	4643 (53.0)	203 (21.5)	< 0.001
*Complications*			
Pneumonia	670 (7.6)	52 (5.5)	0.19
Mechanical ventilation > 96 h	165 (1.9)	24 (2.5)	0.45
Thromboembolic (aggregate)	282 (3.2)	17 (1.8)	0.10
Cardiac (aggregate)	149 (1.7)	22 (2.4)	0.51

Variables reported as count and percentage

*DR* delayed resection, *LOS* length of stay, *IR* immediate resection

**Fig. 5:**
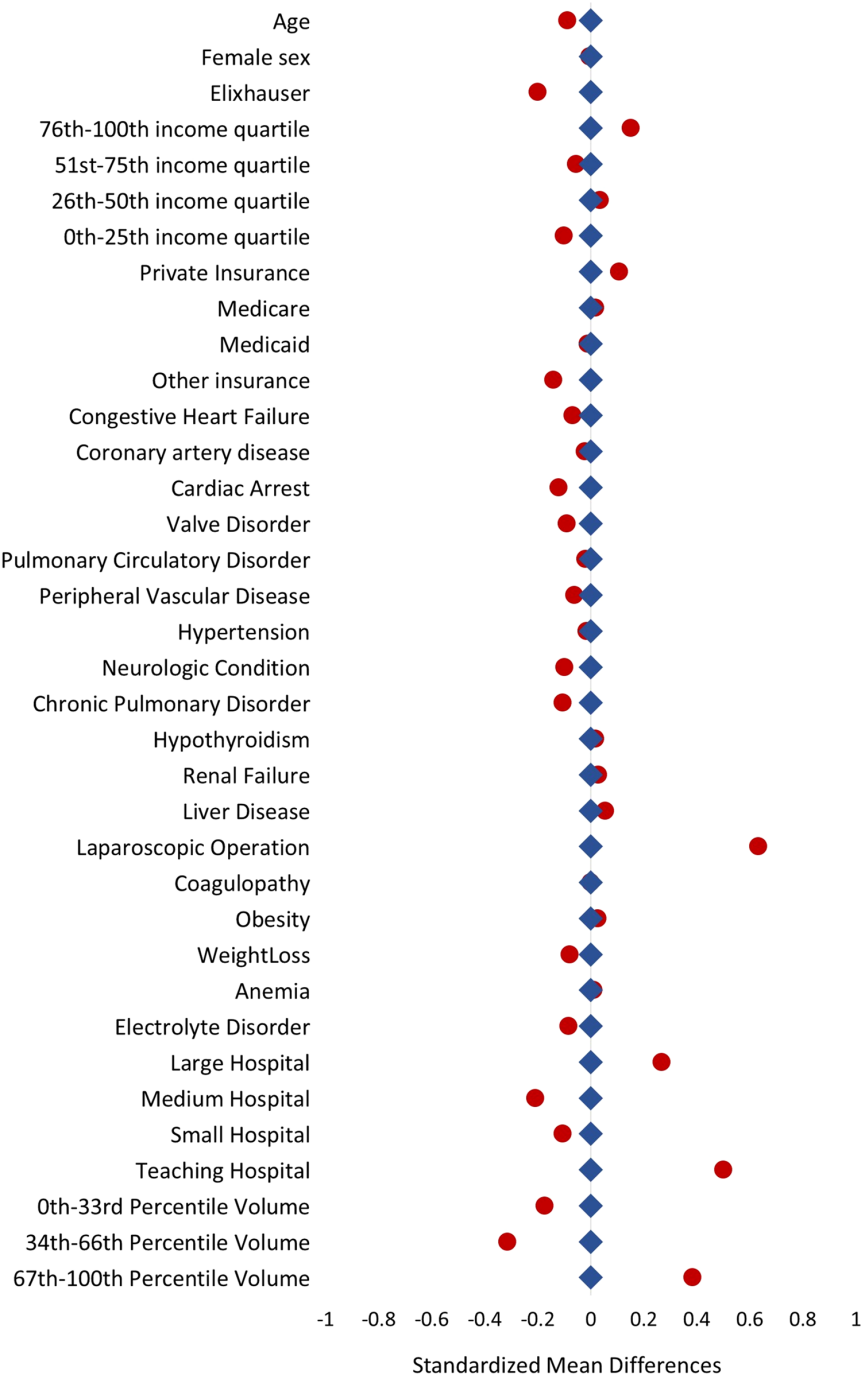
Comparison of standardized mean differences for IR and DR group characteristics before (red) and after (blue) after entropy balancing. DR, delayed resection; IR, immediate resection (Color figure online)

Risk adjustment using entropy balancing produced a well-balanced distribution of covariates between the two groups (Fig. 5). Delayed resection was associated with 0.35-fold adjusted odds (95% CI 0.27–0.41) of receiving an ostomy, relative to IR. There was no association between management strategy and mortality or complications (Table 3).

**Table 3:** Impact of colonic stenting on risk-adjusted outcomes

Outcome	Non-entropy balanced	*P*-value	Entropy-balanced	*P*-value
In-hospital mortality	0.36 (0.16, 0.77)	0.009	0.46 (0.21, 1.01)	0.05
Ostomy creation	0.24 (0.18, 0.33)	< 0.001	0.34 (0.24, 0.46)	< 0.001
*Complications*				
Pneumonia	0.70 (0.41, 1.20)	0.19	0.88 (0.50, 1.57)	0.67
Mechanical ventilation (> 96 h)	1.34 (0.62, 2.89)	0.45	2.20 (1.00, 4.82)	0.05
Thromboembolic (aggregate)	0.55 (0.26, 1.14)	0.11	0.57 (0.26, 1.22)	0.15
Cardiac (aggregate)	1.42 (0.50, 4.00)	0.51	2.00 (0.69, 5.80)	0.20

Each estimate was derived from a separate generalized linear model with estimates for delayed resection relative to immediate resection. Estimates are reported before and after entropy balancing, which adequately adjusted for differences in the two groups (Fig. 5). Outcomes are reported as odds ratios with 95% confidence interval

### Power analysis for primary outcome

Based on prior literature, we assumed a 40% baseline ostomy rate for those admitted with a malignant large bowel resection and undergoing colectomy. At an *α* = 0.05 and *β* = 0.20, to detect a 20% relative reduction in stoma rates, assuming a standard deviation of 15%, a total of 296 patients would be necessary in each group. Given the group sizes of 8,764 in the IR and 943 in the DR, we had sufficient sample size to detect our pre-specified clinically significant difference.

## Discussion

Surgical management of malignant large bowel obstruction remains a challenging clinical entity and is associated with a host of potential systemic and organ-specific complications [5]. Colonic decompression has been suggested as a bridging method to definitive surgical management in order to allow for patient optimization. To our knowledge, the present work represents the first and largest national study comparing outcomes of patients bridged to resection with colonic stenting to those undergoing immediate colectomy. We found a significant increase in the use of colonic stenting as a bridge to resection between 2010 and 2016 from 7.7 to 16.4%. While colonic stenting was associated with lower odds of receiving an ostomy, in-hospital mortality and complications were similar to an immediate resection strategy. Several of these findings warrant further discussion.

A multitude of factors including lack of bowel preparation, malnutrition, and hemodynamic compromise contribute to high risk of complications associated with operations for LBO [21]. Colonic stenting has been proposed as an alternative to urgent resection or surgical diversion in select patients with bowel obstruction due to tumors in the left or sigmoid colon. Successful stenting facilitates rapid colonic decompression, allowing for a less urgent or elective resection [22]. Importantly, this strategy provides a time window for bowel preparation and correction of metabolic derangements [14]. Given the degree of decompression achieved by colonic stenting, operations may be safer and allow for the use of minimally invasive techniques, potentially obviating the need for diversion [22]. In the present analysis, we found colonic stenting as a bridge to resection to be utilized in nearly 10% of all malignant LBO cases. On risk-adjusted analysis, the odds of having a stoma were approximately one-third in the delayed resection group compared to immediate. Interestingly, operations performed within 0–2 days of colonic stenting carried a higher rate of stoma creation, perhaps attributable to unsuccessful stenting, inadequate decompression or perforation [12, 23]. The noted reduction in stoma creation in primary operations over the study period likely reflects the evolution of operative techniques and an improved understanding of risk factors for anastomotic leaks in recent years. Strategy aside, stoma creation is associated with significantly reduced quality of life and mandates a second reversal operation in some cases [24]. With this point in mind, colonic stenting may be a suitable alternative to immediate resection and reduce the need for diversion.

An interesting finding of the study is the similar risk-adjusted odds of in-hospital mortality and complications noted between the DR and IR groups. The two cohorts were comparable in regards to age and comorbidities that were captured in the database. We used entropy balancing to mitigate the effects of bias when comparing the two management strategies [19, 20]. While unadjusted mortality was nearly three times higher in the IR group, this difference was no longer significant on risk-adjusted analysis. Increased multidisciplinary expertise at centers that employ colonic stenting, an advanced endoscopic procedure, as well as potentially higher acuity in patients undergoing immediate resection may explain such findings. On the other hand, patients warranting a resection shortly after stent placement may represent a group with complications such as perforation and may be at increased risk of mortality. In a study of patients in the state of New York, Dolan and colleagues evaluated the outcomes 139 propensity-matched pairs with malignant LBO and found a similar risk of procedural complications between immediate resection and those bridged to resection using colonic stenting [25]. Importantly, in the present work, we found that preoperative stenting allowed for a nearly threefold increase in use of laparoscopic technique for colonic resection. Minimally-invasive approaches, when safe, are often preferred by patients, reduce postoperative pain, and rates of ileus and wound complications [26, 27]. Taken together, our results point to the relative safety of colonic decompression prior to definitive surgical resection in patients who are otherwise deemed candidates for stenting.

While the present study focused on colonic stenting as a bridge to surgery, diverting ostomy followed by definitive resection with or without ostomy reversal represents an alternate management strategy. In a cohort of 443 patients with obstructive left-sided colon cancer undergoing stenting or diverting ostomy as a bridge to resection, survival and locoregional recurrence rates were comparable in both groups [28]. However, the authors found an initial strategy of diverting ostomy to be associated with greater post-resection stomas and subsequent interventions, but lower resection-related complications. As clinical equipoise currently exists for both bridging strategies, treatment should be individualized based on patient and institutional factors noting that initial stenting may lead to avoidance of an ostomy. It is likely that avoidance of an ostomy, when clinically appropriate, is preferred by most patients and results in improved quality of life measures. Further studies comparing bridging strategies in contemporary cohorts or a randomized controlled trial may better delineate the optimal bridging strategy.

This study has several limitations inherent to its design and the structure of the NRD. We limited our analysis to patients admitted with left or sigmoid colon malignant large bowel obstruction, as this population is more amenable to colonic stenting. Limited information regarding clinical factors, including tumor size and cancer stage, are available in the NRD. For the delayed resection group, analysis was only performed for patients bridged to resection with colonic stenting, rather than all patients receiving colonic stents, to ensure appropriate comparison with the immediate resection group. To ensure comparability of the immediate resection to the delayed resection group, patients who carried a diagnosis of bowel perforation or ischemia and underwent immediate resection were excluded, as these patients would not be candidates for colonic stenting. Due to the structure of NRD, follow-up time is limited to a single calendar year and, as such, we could not evaluate ostomy takedown rates or measures of resource use, such as cumulative costs, length of stay, or readmission events. Similarly, quality of life measures, oncologic outcomes, and long-term follow-up data are not available in NRD, limiting our outcome assessment to inpatient measures. Nonetheless, our study includes the largest, nationally representative sample of patients with malignant large bowel obstruction and reports on practice patterns and real-world outcomes of colonic stents as bridge to resection compared to immediate resection.

In conclusion, we found greater use of colonic stenting as a strategy to bridge patients to resection for those admitted with malignant, left-sided large bowel obstruction. Compared to immediate resection, bridging was associated with similar inpatient mortality and morbidity, but significantly reduced rates of ostomy formation as well as greater use of laparoscopic surgery. Among patients who underwent colonic stenting, those undergoing resection within 2 days had ostomy formation rates similar to those undergoing immediate resection. These findings support the relative safety of colonic stenting as a bridge to resection for malignant large bowel obstruction when clinically safe and feasible.

## Supplementary Information

Below is the link to the electronic supplementary material.

Supplementary file1 (DOCX 14 KB)
