# Supplementary material for: Preoperative stents for the treatment of obstructing left-sided colon cancer: a national analysis
Source: Surg Endosc. 2022 Oct 11;37(3):1771–80. doi: 10.1007/s00464-022-09650-8 (PMC10017588; doi:10.1007/s00464-022-09650-8)
Supplement: Supplementary file 1 — Supplementary file1 (DOCX 14 KB) [file 464_2022_9650_MOESM1_ESM.docx]

**Supplemental Table 1.** International Classification of Diseases 9^th^ and 10^th^ Revision diagnosis and procedure codes used to identify study cohort and ascertain complications.

| **Variable** | **ICD-9** | **ICD-10** |
| --- | --- | --- |
| Intestinal Obstruction | 560.89, 560.9 | K56.69, K56.60 |
| Colorectal Cancer | 15.32, 15.33, 15.40 | C18.6, C18.7, C18.7, C19 |
| Resection | 45.75, 45.76, 17.35, 17.36 | 0DBG0ZZ, 0DBG3ZZ, 0DBG4ZZ, 0DBG7ZZ, 0DBG8ZZ, 0DBGFZZ, 0DBM0ZZ, 0DBM3ZZ, 0DBM4ZZ, 0DBM7ZZ, 0DBM8ZZ, 0DBMFZZ, 0DBN0ZZ, 0DBN3ZZ, 0DBN4ZZ, 0DBN7ZZ, 0DBN8ZZ, 0DBNFZZ, 0DTG0ZZ, 0DTG4ZZ, 0DTG7ZZ, 0DTG8ZZ, 0DTGFZZ, 0DTM0ZZ, 0DTM4ZZ, 0DTM7ZZ, 0DTM8ZZ, 0DTMFZZ, 0DTN0ZZ, 0DTN4ZZ, 0DTN7ZZ, 0DTN8ZZ, 0DTNFZZ |
| Stent | 46.86, 46.87 | 0D7E0DZ, 0D7E3DZ, 0D7E4DZ, 0D7E7DZ, 0D7E8DZ, 0DHE0DZ, 0DHE3DZ, 0DHE4DZ, 0DHE7DZ, 0DHE8DZ, 0DHP0DZ, 0DHP3DZ, 0DHP4DZ, 0DHP7DZ, 0DHP8DZ, 0D7F0DZ, 0D7F3DZ, 0D7F4DZ, 0D7F7DZ, 0D7F8DZ, 0DHD0YZ, 0DHD3YZ, 0DHD4YZ, 0DHD7YZ, 0DHD8YZ, 0D7G0DZ, 0D7G3DZ, 0D7G4DZ, 0D7G7DZ, 0D7G8DZ, 0D7H0DZ, 0D7H3DZ, 0D7H4DZ, 0D7H7DZ, 0D7H8DZ, 0D7K0DZ, 0D7K3DZ, 0D7K4DZ, 0D7K7DZ, 0D7K8DZ, 0D7L0DZ, 0D7L3DZ, 0D7L4DZ, 0D7L7DZ, 0D7L8DZ, 0D7M0DZ, 0D7M3DZ, 0D7M4DZ, 0D7M7DZ, 0D7M8DZ, 0D7P0DZ, 0D7P3DZ, 0D7P4DZ, 0D7P7DZ, 0D7P8DZ, 0D7N0DZ, 0D7N3DZ, 0D7N4DZ, 0D7N7DZ, 0D7N8DZ |
| Stoma | 46.10, 46.11, 46.13, 46.20, 46.21, 46.22, 46.23, 46.3, 46.31, 46.32, 46.39 | 0D1H0Z4, 0D1H4Z4, 0D1H8Z4, 0D1K0Z4, 0D1K4Z4, 0D1K8Z4, 0D1L0Z4, 0D1L4Z4, 0D1L8Z4, 0D1N0Z4, 0D1N4Z4, 0D1N8Z4, 0D1B0Z4, 0D1B4Z4, 0D1B8Z4 |
| Pneumonia | 481, 482, 483, 484, 485, 486, 997.31, 997.32, 513.0 | J13, J14, J15, J16, J17, J18, J95.851, J85.0, J85.1 |
| Prolonged Mechanical Ventilation | 96.72 | 5A1955Z |
| Cardiac Complication (aggregate) | 427.1, 427.4, 427.5, 423.3 | I31.4, I46, I47.2, I49.0, I97.12, I97.711 |
| Thrombotic Complication (aggregate) | 453.2, 453.3, 453.4, 453.82, 453.84, 453.85, 453.86, 453.87, 415.1 | I82.4, I82.62, I82.A1, I82.B1, I82.C1, I82.210, I82.220, I82.290, I82.3, I26.02, I26.09, I26.92, I26.93, I26.94, I26.99 |
